# Major biliovascular injury associated with cholecystectomy with the need for percutaneous arterial revascularization and staged right hepatectomy: case report

**DOI:** 10.1590/0102-672020190001e1493

**Published:** 2020-05-18

**Authors:** Pablo Ignacio San Martin FERRADA, Héctor Fabio Losada MORALES, Jorge Alberto Silva ABARCA, Paula Inés Flores MUÑOZ

**Affiliations:** 1 Departamento de Cirugia, Anestesia y Traumatologia, Universidad de la Fontera, Temuco, Region de la Araucania (IX), Chile

**Keywords:** Hepatectomy, Cholecystectomy, Vascular system injuries, Colecistectomia, Hepatectomia, Lesão do sistema vascular

## INTRODUCTION

The cholecystectomy is currently one of most frequently performed surgeries. The incidence of secondary bile duct injury occurs between 0.2-0.3% in the open technique, while for the laparoscopic is 0.5-0.8%^7^ ^,^ ^9^. In terms of the iatrogenic vascular injuries associated with those of the bile duct, there are reports between 12-39%^1^ ^,^ ^4^ ^,^ ^5^. Such injuries worsen the patient›s prognosis and complicate their management. The best resolution for these has not yet been defined.

Among the vascular injuries associated with the cholecystectomy, almost 90% are in right hepatic artery. There are rare vascular injuries associated with bile duct injury that include the hepatic artery proper, the common hepatic artery, the main trunk of the portal vein, the right branch of the portal vein or a major venous injury associated with injury of the right hepatic artery. The literature contains few reports of these cases of major biliovascular injury.

Hepatic ischemia with hepatic parenchymal infarction occurs frequently and may require liver resection or transplant. Death associated with this type of injury is close to 50%^2^ ^,^ ^8^.

## CASE REPORT

Fifty-six old male with history of high blood pressure, gastroesophageal reflux, depression and hypothyroidism consulted for abdominal pain in the upper right quadrant of 20 days’ duration that had intensified in the previous two days, adding nausea and vomiting. He was admitted hemodynamically stable, afebrile, with diagnosis of mild lithiasic acute cholecystitis. He was operated laparoscopically, where a complex cholecystectomy was described with laceration of the right portal vein; therefore, the operation was converted to open laparotomy, with bleeding of up to three liters. A red blood cell transfusion was indicated. The bleeding was brought under control, and bile filtration was observed, which is why the bile duct was explored and a Strasberg E4 bile duct injury was described^7^, which was managed with two nelaton probes to the duodenum. The patient was moved to critical patient unit after surgery, where he recovered, and afterwards referred to liver, pancreatic and biliary surgical team of Hospital Hernán Henríquez Aravena. He was admitted hemodynamically stable. 

**FIGURE 1:**
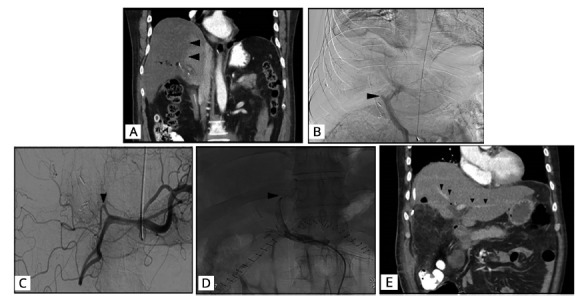
A) Ischemia of right hepatic lobe; B) partial thrombosis right hepatic vein; C) section hepatic artery proper; D) recanalization left hepatic artery; E) effective arterial perfusion of hepatic remnant

Computed tomography of abdomen and pelvis (Figure 1A) suggested right hepatic ischemia. A vascular study was proposed, and angiography showed partial thrombosis of the right portal vein (Figure 1B) and complete stenosis of the hepatic artery proper (Figure 1C). Ischemia of the right hepatic lobe and left hemi-liver with irrigation originating from the left gastric artery and gastroduodenal artery was described. Endovascular reperfusion of the hepatic artery proper was performed with a balloon, achieving re-permeabilization of the left hepatic artery (Figure 1D). The patient evolved with sepsis and liver failure; magnetic nuclear resonance of the abdomen was performed with images suggestive of infarction and infected collection in the right hemi-liver, which was managed with percutaneous drainage. Due to the persistence of sepsis, despite draining the collection, it was decided to perform a right hepatectomy, finding complete necrosis of the right hemi-liver and an inflammatory mass that involved the retroperitoneum, which made dissection of the inferior vena cava difficult. A hepaticostomy was left on the stump of the left hepatic duct, and the anastomoses were deferred due to local conditions and the patient’s condition. He evolved with persistence of cholestasis, verifying malfunction of the hepaticostomy. Therefore, the decision was made to perform a terminolateral hepaticojejunostomy. The patient evolved with a low-debit biliary fistula with no dilation of the bile duct, and percutaneous drainage was carried out. He became hemodynamically stable, with persistence of hepatic dysfunction. Duplex ultrasound and computed tomography of the abdomen showed a hepatic remnant with adequate arterial and venous perfusion, with no biliary obstruction (Figure 1E). The cholestasis reduced gradually and the patient was discharged in good condition at 38^th^ postoperative day.

## DISCUSSION

Despite the cholecystectomy being common in our environment, the bile duct injury with associated vascular injury continues to be present, and this was an extreme case that required multidisciplinary management.

The literature contains few reports of major biliovascular injuries; in most cases they are associated with hepatic ischemia or hepatic dysfunction, which may require a liver resection or transplant. The mortality rate associated with such injuries is close to 50%^8^.

In some cases of this type of injury, hepatic resection with reconstruction of the bile duct has been preferred over the liver transplant ^3^ ^,^ ^10^. There is a report of a portal vein injury discovered intra-operatively, where hepatectomy and portosystemic derivation were proposed, with subsequent liver transplant from a donor corpse^11^.

In this case, we faced the challenge of a right portal vein injury with hemi-liver ischemia and injury of the hepatic artery proper with involvement of the left hepatic artery. Endovascular revascularization of the hepatic artery proper was very important to ensure the flow to the left hepatic artery.

Other challenge was to define the point to perform the hepatic resection on a patient with liver ischemia and necrosis with risk of developing infection and sepsis.

With the few cases that exist in the literature, it is impossible to determine the superiority of the early hepatectomy over the deferred hepatectomy. In this case, achieving reperfusion of the left hepatic artery was very important to obtain adequate perfusion of the post-hepatectomy remnant.

During evolution and programming the liver resection, the patient developed infection in the right hemi-liver with necrosis. The initial treatment was drainage of the collection and antibiotic therapy, with no improvement; therefore, it was decided to perform an emergency hepatic resection. The hepaticostomy malfunctioned, producing cholestasis despite the improvement in the patient’s organic dysfunction; then, decision was to perform a Roux-en-Y hepaticojejunostomy anastomosis. In this case, as in all cases of bile duct reconstruction, we considered the use of magnification and microsurgical technique fundamental^6^.

After the biliary reconstruction and persistence of cholestasis, imaging is very important to ensure an adequate perfusion for remnant liver and to rule out biliary complications.
